# Effectiveness of manual therapy, computerised mobilisation plus home exercise, and home exercise only in treating work-related neck pain: study protocol for a randomised controlled trial

**DOI:** 10.1186/s12891-022-06093-z

**Published:** 2022-12-22

**Authors:** Weiming Wang, Chang Ji, Lars Louis Andersen, Yafei Wang, Yangyang Lin, Li Jiang, Shuwei Chen, Yangfan Xu, Ziping Zhang, Le Shi, Yuling Wang

**Affiliations:** 1 Rehabilitation Medicine Center, The Sixth Affiliated Hospital, Sun Yat-sen University, Guangdong Province Guangzhou, China; 2 Guangzhou Sport University, Guangdong Province Guangzhou, China; 3 National Research Centre for the Working Environment, Copenhagen, Denmark; 4 Medical College of Rehabilitation, Southern Medical University, Guangdong Province Guangzhou, China

**Keywords:** Computerised cervical mobilisation, Physical therapy, Home exercise, Randomised controlled trial, Chronic neck pain, Manual therapy

## Abstract

**Background:**

Work-related neck pain (WRNP) is a leading cause of disability and absenteeism. Patients with neck pain often have neck muscle tenderness and decreased cervical mobility, which are sometimes combined with psychosocial issues, such as pain catastrophising, thereby reducing their work ability. Whilst multidisciplinary treatments, including pharmacological interventions, manual therapy and specific neck exercises, have produced positive outcomes, effective personalised treatment modalities are still needed. Furthermore, manual therapies using the hands can bring fatigue to therapist. Occiflex is a computerised device that can provide personalised segmental joint mobilisation based on symptoms and injury of the patient and then provide a medium range of joint activities to improve range of cervical motion. This study aims to compare the effect of computerised mobilisation performed with Occiflex with that of traditional manual therapy on WRNP.

**Methods:**

We will conduct a prospective randomised controlled trial including 150 patients with WRNP. These patients will be randomly assigned to one of three groups: (i) home exercise (TE), (ii) home exercise plus Occiflex therapy and (iii) home exercise plus manual therapy delivered by a physical therapist. Ten treatment sessions will be performed in four weeks. During the trial, these patients will receive only the assigned treatment and the standard patient education and will be asked not to use any analgesics unless strictly necessary. Assessments by trained evaluators will occur at baseline, week 4 and week 12. The primary outcome measures will include visual analogue scale (VAS) for pain and neck disability index (NDI) at each time point. Secondary outcome measures will include cervical range of motion (CROM), pressure pain threshold (PPT), global perceived effect (GPE) and sick leave. Group by time differences will be analysed using linear mixed models with repeated measures.

**Discussion:**

This protocol describes the methods for a randomised controlled trial to compare the effectiveness of computerised versus manual mobilisation techniques in treating WRNP. The results will provide an alternative method (Occiflex) that is possibly effective for treating neck pain whilst minimising the manual work done by therapists.

**Trial registration:**

The study protocol was retrospectively registered at http://www.chictr.org.cn (registration number: ChiCTR2100053076) on November 10, 2021.

## Background

Work-related neck pain (WRNP) is a leading cause of disability and absenteeism in our society [1]. Such condition is highly prevalent amongst office workers, which may be partly due to their working environment, prolonged working hours and psychosocial factors [2, 3]. Sedentary work for extended periods of time may result in excessive neck muscle activity and fatigue [4], whilst poor postural habits [3] and maladaptive motor control [5] contribute to the development of pain and fatigue of the neck muscles. The origin of WRNP is likely multifactorial, especially in the case of chronic pain. Patients with WRNP show a range of symptoms, including neck stiffness, upper extremity pain, headache and, in some cases, psychological problems, such as depression, anxiety and pain catastrophising [6]. These problems not only pose a burden for individual workers but also lead to substantial costs for the whole society [1].

Amongst the conservative treatments for neck disorders, physical therapy is widely used in clinical practice. Exercise and manual therapy are recommended for treating many types of neck pain [7] and remain a priority for researchers. Several studies have proven that exercise can improve pain and disability in chronic non-specific neck pain [8, 9]. For instance, Andersen et al. found that one year-physical exercise intervention can improve the overall pain perception of office workers with chronic musculoskeletal pain, indicating a significant decrease in central sensitisation [10]. Mobilisation of the spine and other manual therapies have also shown promising effects yet inconclusive results according to several meta-analyses [11–13]. Though these techniques have been reported to elicit hypoalgesia and reverse pain sensitisation [14], the manual force applied by physical therapists during cervical mobilisation is largely inconsistent [15, 16]. In practice, having the same therapist repeat the techniques with precision and consistency over time can be challenging partly because of the manual technique or the therapist’s fatigue. Moreover, practitioners show some differences in the manual techniques they apply in each therapeutic session (i.e., manipulation and mobilisation). In addition, spinal manipulation has been reported to cause mild to moderate adverse effects when performed on the upper spine possibly due to the inconsistencies in the technique and manually applied force. Manual therapy is widely used by physical therapists yet should be applied with caution especially during the administration of high-velocity or aggressive techniques [17].

To overcome the inherent disadvantages and safety issues related to manual therapy, researchers have developed a device capable of 3D computerised neck mobilisation. This device, called Occiflex™, enables therapists to implement a hands-free, sustained mid-range neck mobilisation for patients. This device comprises an adjustable bed and a cradle that is capable of any movement in a 3D space with six degrees of freedom [18]. Before the treatment process, the therapist should teach the device a series of individualised mobilisations for each patient based on their impairment. Subsequently, the Occiflex can carry out the recorded mobilisation automatically and precisely in a slow and smooth manner whilst avoiding the end of the available neck range of motion. Pilot studies have examined the safety of computerised mobilisation and revealed that the continuous and accurate mid-range mobilisation of the cervical spine can reduce neck muscle contraction, alleviate pain and increase cervical range of motion (CROM) [18–20]. River et al. explained that patient-reported improvements may be associated with reduced afferent pain and diminished central sensitisation following the computerised mobilisation. However, no randomised study has compared the effects of computerised mobilisation with those of manual therapy on WRNP.

### Objective

The objective of this article is to introduce a protocol for comparing the effects of computerised mobilisation performed with Occiflex with those of traditional manual therapy on WRNP. By adding these two mobilisation techniques to a standard exercise programme, this article also determines whether computerised mobilisation or manual therapy produces more benefits than pure exercise intervention. Computerised mobilisation is assumed to be superior to manual therapy and generate more effects than exercise alone.

## Methods/design

A prospective, three-armed, open-label randomised controlled trial of patients with WRNP will be conducted in the out-patient physiotherapy department of the Sixth Affiliated Hospital, Sun Yat-sen University.

### Ethics approval

The study design and procedures were approved by ethics committee of the Sixth Affiliated Hospital, Sun Yat-sen University (Protocol Number: 2021ZSLYEC-317) and qualified for registration in the Chinese Clinical Trial Registry (ChiCTR2100053076). All participants must provide their informed written consent prior to their enrolment in the study.

### Sample selection

**Fig. 1:**
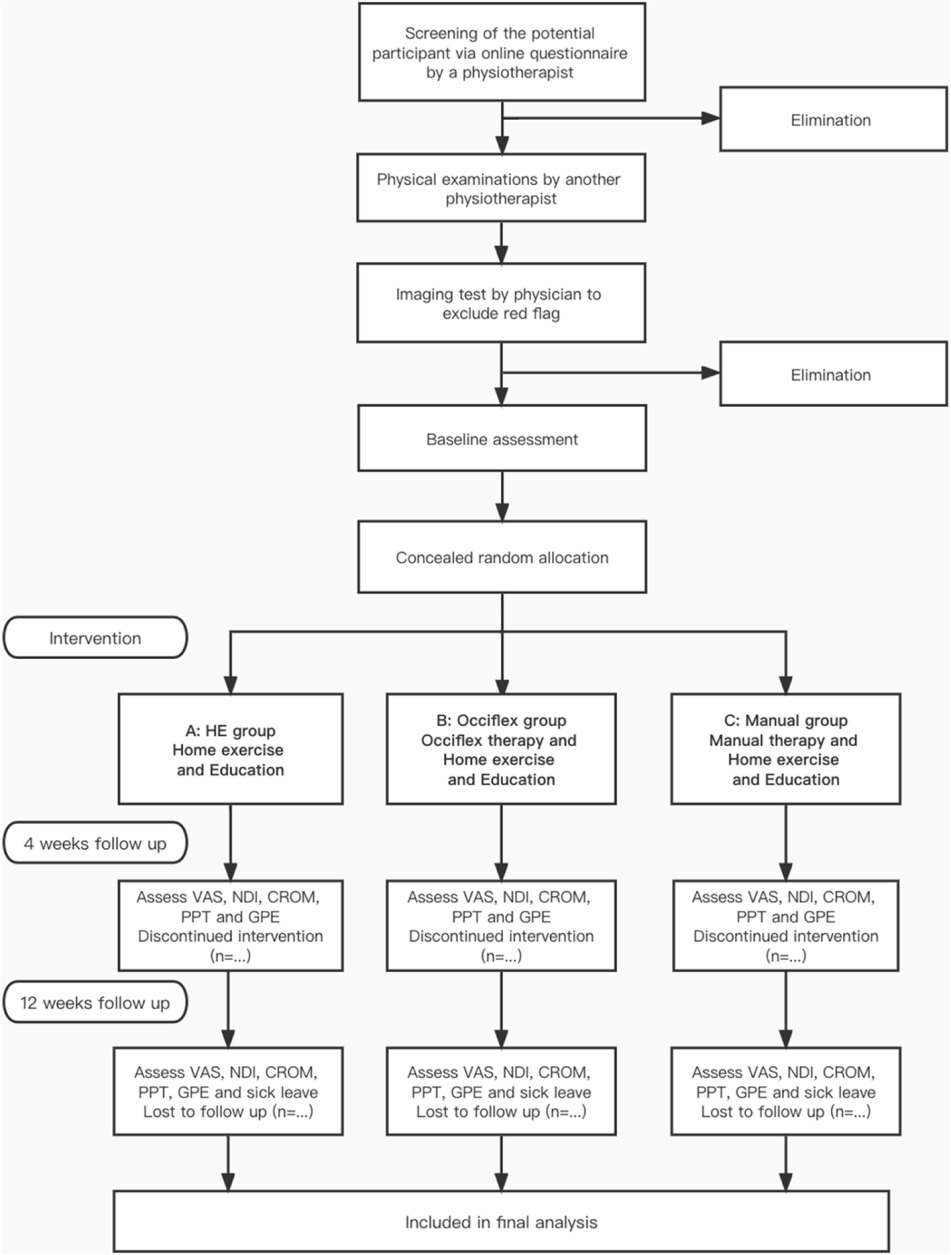
Flowchart of research protocol

Individuals with WRNP will be screened via an online questionnaire by a physiotherapist and will be selected based on the eligibility criteria [4, 5] listed in Table 1. Before enrolment, each participant should undergo a physical assessment, including medical history, neurological test and special testing, to be administered by another therapist. Those who pass the preliminary screening would be further examined by a physician to exclude definite pathological factors, such as tumours and fractures, via an X-ray imaging test. Figure 1 shows the flowchart of the research protocol.

**Table 1:** Inclusion and exclusion criteria

Inclusion criteria
**• **Age: 18 and 55 years • Office workers performing at least 4 h of computer work per day (on average) • Current neck pain, with or without pain in head or arm region • Experiencing pain for least 3 months, at a moderate or high pain intensity (≥ 5 of 10, Visual Analog Scale) • No cognitive impairment and volunteer to participate in the study
Exclusion criteria
• Pain caused by definite pathological factors, such as cerebrovascular diseases involving vertebral arteries, spinal cord pathology, cervical cancer or fracture • Neck pain secondary to diseases such as rheumatoid arthritis, ankylosing spondylitis and cervical spine infection • Suffering from cervical myelopathy or radiculopathy, coupled with motor, reflex, and/or sensory changes in the upper limb • Recurrent vertigo, or dizziness • Previous surgery to the cervical spine

### Randomisation

After completing the baseline assessment, the eligible subjects will be randomised in equal portions to the home exercise group, Occiflex group and manual therapy group via a computer-generated block allocation with a block size of 6 or 9. Randomisation will be stratified by the baseline score (≤ 25 vs. ≥26 on a 0–50 scale) on the neck disability index (NDI) and balanced by gender in a 2: 1 ratio (female vs. male) for each group. This procedure ensures that the participants with the same severity of neck disability will be allocated to each group and that all groups are comparable in sex. Both the entry process and group allocation are unblinded to the researchers, examiners and participants.

### Interventions

All participants can only receive the assigned treatment and a standard education.

booklet during the course of the study. They will be asked not to use any analgesics during the trial unless strictly necessary. All medication intake will be reported, and exclusion will be considered if a drug is taken for more than three days after enrolment. The participants will be asked to report any adverse event during the treatment period.

#### Group a (HE group): Home exercise + Booklet education

**Fig. 2:**
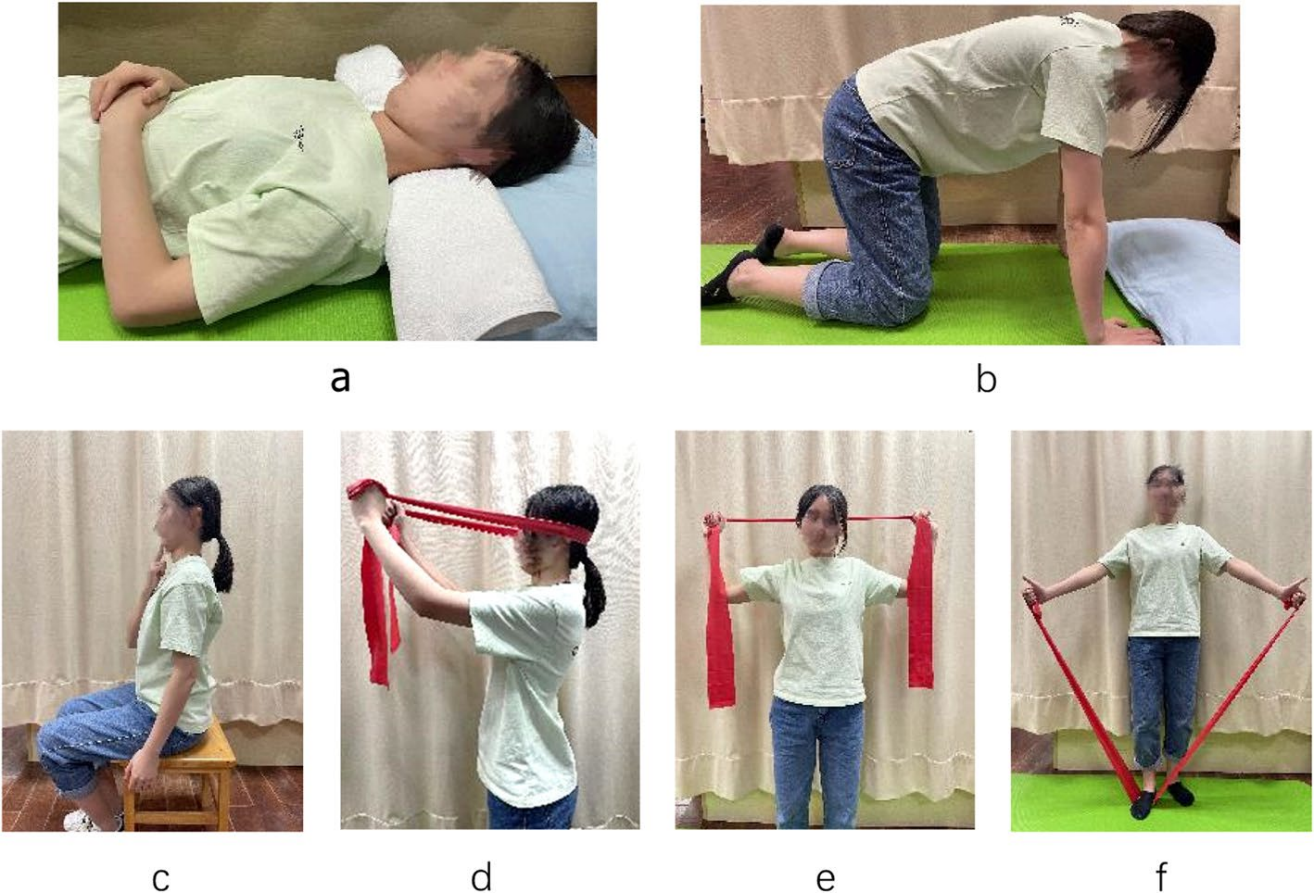
Standard therapeutic exercise program. **a** Cranio-cervical flexion in supine position; (**b**) Cranio-cervical flexion in Quadrupedal position; (**c**) neck movement and cranio-cervical flexion in sitting position; (**d**) Co-contraction deep and superficial neck flexors with Thera-band; (**e**) exercise for lower trapezius and scapular muscles with Thera-band; (**f**) Resisted shoulder elevation with Thera-band

The participants in the HE group will be instructed to carry out a standardised home exercise programme regularly five times a week for four weeks on their own. The protocol will be taught to these participants during the first week and will be reinforced by a physiotherapist during their visit to the department. The actual amount of time will be recorded in a training diary and checked at each visit. Those participants who perform the home exercises less than three times a week will be withdrawn from this study. All participants will also receive booklet education on different topics, including office ergonomics and recommended neck exercises. The exercise prescriptions are listed in Table 2  [5, 21]. Figure 2 shows the standard home exercise programme using Thera-band in different positions.

**Table 2:** List of exercises and prescriptions in the standard home exercise program

Exercise	Position	Description
1	Supine	Cranio-cervical flexion (CCF) with a towel on the back of neck for 10s, 10 repetitions with 10s rest, 3 sets totally (Fig. 2a)
2	Quadrupedal	Neck in neutral position, perform the cranio-cervical flexion and maintain the posture for 10s, 10 repetitions with 10s rest, 3 sets (Fig. 2b)
3	Sitting	Move neck into flexion, extension, side flexion and rotation to each side. Then perform cranio-cervical flexion for 10 repetitions and each with 10s of contraction (Fig. 2c)
4	Sitting	Co-contraction deep and superficial neck flexors with Thera-band for 10 repetitions, 3 sets totally (Fig. 2d)
5	Sitting	Co-contraction exercise for lower trapezius and scapular muscles with Thera-band for 10 repetitions, 3 sets totally (Fig. 2e)
6	Standing	Resisted shoulder elevation exercise in scapular plane with Thera-band (Fig. 2f)

Notes: Participants will be given the exercise instructions within different sessions (Session 1-2: Exercise 1 and 2; Session 3-6. Exercise 1, 2, 3 and 4; Session 7-10. Exercise 1, 2, 3, 4, 5 and 6). Each session of exercises should not last more than 30 minutes to avoid fatigue

#### Group b (Occiflex group): Occiflex + Home exercise + Booklet education

**Fig. 3:**
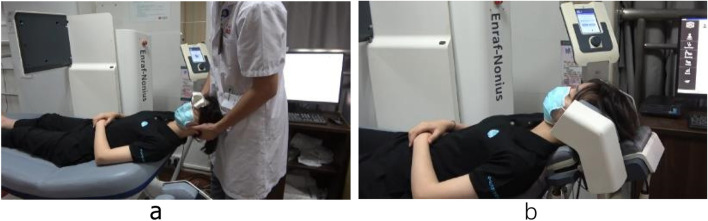
Computerized mobilisation using the Occiflex device. **a** Physiotherapist teaching the device individualized mobilisations; (**b**) Occiflex repeat the recorded mobilisation automatically

Patients in the Occiflex group will receive the standard home exercise programme and health education as group A combined with the computerised mobilisation (Occiflex) treatment. The Occiflex device can record any head and neck mobilisation in a 3D space as performed by the physiotherapist (Fig. 3a). The recorded mobilisation will be used as a template for repeated movement (Fig. 3b). The participants allocated to the Occiflex group will be divided into three categories according to their symptoms and a physical examination of their neck pain [7]: (a) neck pain with mobility deficits; (b) neck pain with headache (cervicogenic); and (c) neck pain with shoulder radiating pain (radicular). Individualised mobilisation will be determined for each category following three principles [18]: (a) stretching shortened or tight muscles; (b) mobilisation of facet joints relevant to pain; and (c) extending the limited range of motion based on the CROM examination. This mobilisation programme will be determined by an experienced physiotherapist during the first visit and performed using a computer afterwards. Each treatment session will last for 20 min and will be done every 3 days for a total of 10 times in 1 month.

#### Group c (Manual group): Manual therapy + Home exercise + Booklet education

**Fig. 4:**
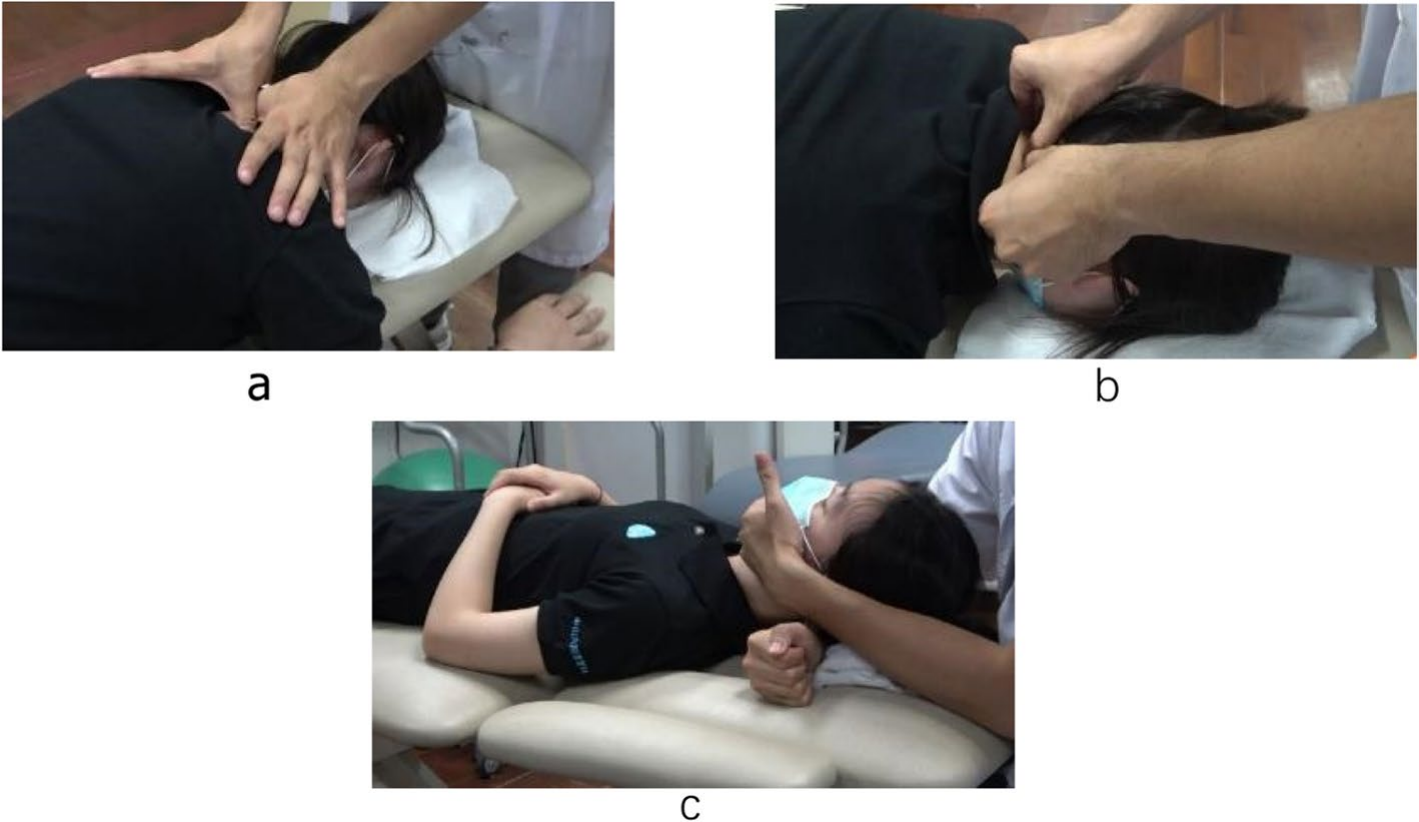
Manual therapy protocol. **a** Thoracic intervertebral joint mobilisation; (**b**) Cervical articular joint mobilisation; (**c**) Suboccipital muscle stretching

The participants in the manual group will receive the standard home exercise programme and health education combined with manual therapy based on the neck pain clinical practice guidelines. Similar to Group B, all participants in this group will be divided into three categories and treated with cervical and thoracic mobilisation accordingly [7]. The physiotherapist who performs manual therapy must be qualified and should have received the standardised training as described in *Maitland’s Vertebral Manipulation (7th edition)*. Each treatment will last for approximately 20 min and will be performed every 3 days for 10 times in 1 month. The manual therapy technique [21–23], which consists of three parts, is illustrated in Fig. 4.

- Thoracic intervertebral joint mobilisation. The patient lies prone with his/her forehead resting on his/her hands. The therapist will place a pad of his/her thumbs in a spinous process to rhythmically apply pressure to the vertebra from T1 to T4 separately, usually in a posterior-to-anterior direction. The thumbs may be positioned tip to tip or with the tips side by side to transmit the oscillating pressure from body weight to the hand. This mobilising technique will be carried out 2 min for each intervertebral joint.
- Cervical articular joint mobilisation. The physiotherapist will palpate the neck to find the two or three most dysfunctional joints from C2 to C7 and then use his/her thumbs to perform passive joint mobilisations to those joints. The oscillatory pressure will be directed postero-anteriorly against an articular process at a speed of 1 to 2 Hz for 3 min each according to irritability of the patient’s disorder.
- Suboccipital muscle stretching. The patient lies supine with his/her head in a neutral position. The physiotherapist places one supinated forearm underneath the patient’s neck with the other hand fixing the chin. The contact forearm then pronates against the patient’s occiput and sustains the force for 30 s to stretch the suboccipital muscle. This technique can be repeated 3 times as required.

### Outcome measures

Personal information, including age, gender, BMI, international physical activity questionnaire (IPAQ) [24], working years and time since the onset of symptoms, will be collected at the baseline. The primary outcome measurements will include the VAS for pain and NDI to be administered at weeks 4 and 12. Secondary outcomes will include CROM, pressure pain threshold (PPT), global perceived effect (GPE) and sick leave. These evaluations will be carried out by an evaluator trained with these procedures. The adverse reactions during therapy will also be recorded.

#### Visual analogue scale (VAS) for pain

The participants will be asked to indicate the intensity of their neck pain during the last week on a 10 cm horizonal scale, where 0 means no pain and 10 means unbearable pain [25]. The minimal clinically important change values range from 2.5 to 4.9 points for patients with neck pain, and an improvement below 1.5 points can be interpreted as irrelevant for pain intensity [26]. The VAS will be obtained at the baseline, week 4 and week 12 of the study period (post-treatment and 3 months follow up).

#### Neck disability index (NDI)

The NDI is often used for a self-assessment of the cervical spine function of neck pain patients with validity and reliability [27]. This index consists of 10 questions covering different aspects of the patients’ daily life activities. Each item is rated on a scale of 0 to 5, where 0 means ‘no pain’ and 5 means ‘unbearable pain’. A higher score indicates greater disability. The clinically important difference for NDI is approximately 5 points to demonstrate treatment benefits after intervention [28]. The NDI will be measured at the baseline, week 4 and week 12 of the trial.

#### Cervical range of motion (CROM)

CROM refers to the angle of motion when the neck moves in a certain direction, which is an indicator for assessing the motion of the neck. Cervical range-of-motion device (CROM; Performance Attainment Associates, Lindstrom, MN) provides a clinical tool for accurately measuring CROM, including flexion, extension, side bending and rotation in sitting position, with high intraclass correlation coefficients (ICC) ranged from 0.88 for flexion (95% CI: 0.73–0.95) to 0.96 for left rotation (95% CI: 0.91–0.98) [29]. The CROM will be measured at the baseline, week 4 and week 12 of the study period.

#### Pressure pain threshold (PPT)

The PPT is recorded using the digital algometer FPX-25 (Wagner Instruments, Greenwich, CT) to measure the midpoint of the bilateral trapezius muscle and the spinous process of the vertebra C2 [30]. The therapist gradually increases the pressure until the participant complains of pain or discomfort. PPT has reported high intra- and inter-rater reliabilities (ICC = 0.94–0.97; ICC = 0.79–0.90, respectively) amongst patients with neck pain[31]. The measurement should be repeated three times, and the average scores should be computed. The PPT will be assessed at the baseline, week 4 and week 12 of the study period.

#### Global perceived effect (GPE)

The GPE provides physicians with a reference on the overall recovery condition of their patients. GPE scales are commonly advocated for use in chronic pain research and clinical practice[32]. In this scale, ‘5’ indicates that the neck pain deteriorates to the greatest extent, whereas ‘5’ indicates complete recovery. The GRE will be assessed at weeks 4 and 12 of the study period.

#### Sick leave

Sick leave was measured three months after the baseline by asking the participants: ‘How many times were you absent from work because of neck pain since the last treatment?’ [33]. The length and frequency of sick leaves will be recorded for comparison.

### Sample size calculation

The sample size will be calculated using PASS 15.0.5 based on the means, standard deviations and an alpha of 5% (0.05) with a unilateral contrast. Assuming that the standard deviation of NDI scores is 8, then 38 participants per group will give the study 80% power to detect minimum clinically important differences (MCID) of 5 units in NDI [34]. Meanwhile, 41 participants per group are required based on the 10 cm VAS scale with a standard deviation of 5 and a clinically significant difference of 3 points [26]. To accommodate an expected dropout rate of 20% during follow-up and to have sufficient statistical power for the different variables in this study, a total of 150 participants will be sufficient.

### Statistical analysis

The statistical analysis will be carried out using the IBM SPSS Statistics 26 software. Descriptive statistics will be used to describe the study population and will be summarised per group based on the number of observations, means and standard deviations. The primary and secondary outcome variables (VAS, NDI, CROM, PPT and GPE) will be analysed by intention to treat and by using linear mixed models with repeated measures. The linear mixed model requires the residuals to be normally distributed. The Shapiro–Wilk test will be applied to test the normality. In case of a non-normal distribution of the residuals, an appropriate non-parametric test will be conducted. Multiple post-hoc comparisons will be conducted through Bonferroni’s contrast for parametric distributions. All tests will be two sided, and the level of significance is set to 0.05. The missing data in this study will be handled with a mean imputation or mixed modelling approach.

## Discussion

A previous pilot study reported that a computerised 3D cervical mobilisation device can be safely administered to patients with chronic neck pain. Using a small sample, this pilot study reported that such device can effectively alleviate neck pain and increase CROM [20]. Another preliminary trial investigating the physiological effect of computerised mobilisation reported a positive change in neck posture and cervical neuromuscular control for neck pain after the use of computerised mobilisation. However, neither of these studies compared the effects of computerised mobilisation and manual therapy on chronic neck pain [18]. To fill this gap, the present protocol describes the methods for a randomised controlled trial to compare the effectiveness of these techniques. Results of this study can help lighten the workload of therapists.

This article proposes two types of treatments, namely, manual therapy (mainly joint mobilisation and muscle stretching) and machine-assisted mobilisation based on the symptoms of neck pain. By observing how these two therapies reduce pain and improve functional ability, this article investigates whether Occiflex could be an alternative method that is possibly effective for treating neck pain. A control group with only home exercise and patient education will be added for comparison to understand the addition effect of mobilisation treatment and to gain a better understanding of the mechanism for WRNP. Computerised mobilisation is assumed to be superior to manual therapy and can generate more effects than exercise alone. The study contributes to the development of an evidence-based computerised therapy that can improve the therapeutic techniques being used in this field of clinical practice.

## Data Availability

Data sharing is not applicable to this article as no datasets will be generated or analysed in this study.

## Abbreviations

ANOVA: Analysis of variance
CCF: Cranio-cervical flexion
CROM: Cervical range of motion
GPE: Global perceived effect
ICC: Intraclass correlation coefficients
IPAQ: International physical activity questionnaire
MCID: Minimum clinically important differences
NDI: Neck disability index
PPT: Pressure pain threshold
HE: Home exercise
VAS: Visual analogue scale
WRNP: Work-related neck pain
